# PHLDA2 regulates EMT and autophagy in colorectal cancer via the PI3K/AKT signaling pathway

**DOI:** 10.18632/aging.103117

**Published:** 2020-05-08

**Authors:** Zhan Ma, Shuping Lou, Zheng Jiang

**Affiliations:** 1 Department of Gastroenterology, The First Affiliated Hospital of Chongqing Medical University, Chongqing 400016, P.R. China; 2 Department of Maternal and Child Health, School of Public Health, Tongji Medical College, Huazhong University of Science and Technology, Wuhan 430030, China

**Keywords:** PHLDA2, colorectal cancer, autophagy, apoptosis, tumorigenesis

## Abstract

High levels of the imprinted gene pleckstrin homology like domain family A member 2 (PHLDA2) correlate with tumor progression in several malignancies. Here, we investigated the effects of PHDLDA2 expression in CRC through assays of cellular proliferation, invasion, migration, and apoptosis. We also screened for possible mechanisms of action. Our results show that PHLDA2 was upregulated in CRC tissues. Knockdown of PHLDA2 inhibited cellular proliferation, invasion, migration, and epithelial-mesenchymal transition (EMT) *in vitro*. Knockout of PHLDA2 promoted cellular apoptosis, in part by activating autophagy. PHLDA2 knockout also inhibited tumorigenesis and expression of KI67 protein *in vivo*. The effects of PHLDA2 on autophagy and EMT were mediated in part via the PI3K/AKT signaling pathway. Taken together, these results suggest that downregulation of PHLDA2 inhibits tumor growth and PI3K, thereby promoting autophagy and inhibiting EMT, in part through the PI3K/AKT/mTOR and PI3K/AKT/GSK-3β signaling pathways.

## INTRODUCTION

Colorectal cancer (CRC) is the most common malignancy of the human digestive system. As a cause of cancer-related death, CRC is ranked second only to lung cancer [1]. Therapy is usually less extensive and more successful when CRC is detected early [2]. Therefore, the discovery of new biomarkers would benefit CRC patients [3]. Pleckstrin homology like domain family A member 2 (PHLDA2) is a maternal imprinting gene. The loss of this form of imprinting can result in gene overexpression [4], with tissue-specific differential imprinted gene variation due to mono-allelic expression [5]. PHLDA2 inhibits tumorigenesis and metastasis in osteosarcoma patients via the PI3K/AKT/mTOR pathway [6]. PHLDA2 knockdown restrains invasion and proliferation of breast cancer [7] and inhibits the invasion and migration of pancreatic ductal adenocarcinoma, although the specific mechanisms of action are unclear [8]. Osteosarcoma is a type of mesenchymal malignancy, while breast cancer and pancreatic ductal adenocarcinoma are epithelial malignancies. Herein, we assessed whether PHLDA2 promotes cancer and chose to test this in CRC because it is an epithelial tumor.

Epithelial-mesenchymal transition (EMT) is a process that converts immobile epithelial cells into active mesenchymal cells, which can promote the invasion and metastasis of cancer cells [9]. In addition, PHLDA2 downregulation facilitates EMT via the GSK-3β pathway in osteosarcoma cells [10]. The expression of GSK-3β is regulated by the PI3K/AKT signaling pathway [11].

Autophagy is a lysosomal degradation pathway, which is necessary for survival, differentiation, development, and homeostasis [12]. Previous investigations have demonstrated imprinted genes to regulate autophagy. For example, MEG3 inhibits autophagy and ARHI induces autophagy in bladder and ovarian cancer cells [13, 14]. PHLDA2 (TSSC3) has been reported to promote autophagy in osteosarcoma cells [6]. Autophagy can be regulated by the PI3K/AKT/m-TOR pathway and PHLDA2 has been reported to inhibit such pathway *in vitro* [6]. In addition, PHLDA2 has been shown to activate the PI3K/AKT pathway *in vivo* [15].

A role for PHLDA2 in CRC has not been reported. Therefore, the purpose of this study was to explore whether PHLDA2 affects CRC EMT and autophagy through the PI3K/AKT signaling pathway. We also tested the effects of PHLDA2 on CRC functions and development. Our results may provide a novel diagnostic biomarker and potential therapeutic target for CRC.

## RESULTS

### *In vitro* data

### Levels of PHLDA2 in CRC tissue, HCT116 cells, and SW480 cells

**Figure 1:**
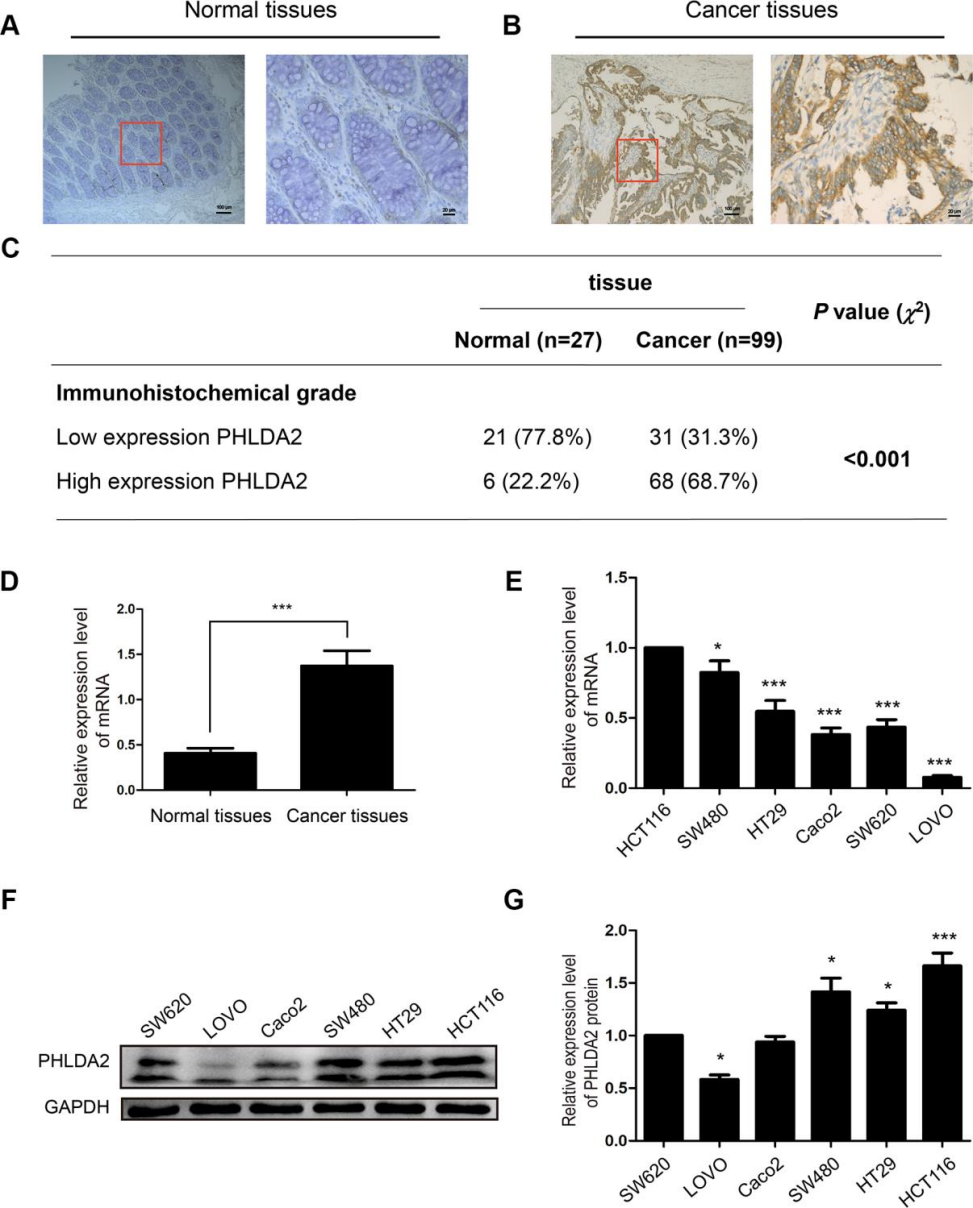
**PHLDA2 expression in CRC tissue, adjacent normal tissue, and cell lines.** (**A**–**C**) Immuno-histochemical staining and analysis of PHLDA2 protein in CRC tissue and adjacent normal tissue (magnification, ×100 and ×400). (**D**) RT-qPCR was used to detect mRNA expression levels of PHLDA2 in 29 CRC tissues and paired normal tissues. (**E**–**G**) RT-qPCR and western blot analyses were used to detect mRNA and protein expression of PHLDA2 in six CRC cell lines. Data are shown as mean ± SD; *P < 0.05, **P < 0.01, and ***P < 0.001.

Protein and mRNA levels in CRC and adjacent tissue were assessed by IHC (CRC tissue, n=99; adjacent tissue, n=27) and RT-qPCR (n=29). Levels of PHLDA2 in CRC tissue were higher than in adjacent normal tissue at both the protein level (χ^2^=18.90, *P* < 0.001, Figure 1A–1C) and mRNA level (*P* < 0.001, Figure 1D). Using WB and PCR, we found that protein and mRNA levels of PHLDA2 were higher in HCT116 and SW480 cells than in the six other CRC cell lines (Figure 1E–1G); therefore, these cell lines were used for subsequent experiments in our study.

### PHLDA2 levels correlate with clinicopathological features

In order to measure the clinical significance of PHLDA2, we investigated the relationships among PHLDA2 expression and clinicopathological characteristics of CRC patients. As shown in Table 1, PHLDA2 expression correlated with lymphatic metastasis (*P* = 0.025) and TNM stage (*P* = 0.009). No difference was found for age, gender, or distant metastasis. These results suggest that PHLDA2 may promote CRC progression.

**Table 1:** Correlations between PHLDA2 expression and clinicopathologic features in 99 colorectal cancer patients.

**Clinicopathological feature**	**PHLDA2 expression**			***χ*^2^**	***P* value**
	**Total**	**Low**	**High**		
	**99**	***n*=31 (31.3%)**	***n*=68 (68.7%)**		
Age (years)					
< 65	59	19 (61.3)	40 (58.8)	0.054	0.817
≥ 65	40	12 (38.7)	28 (41.2)		
Gender					
Male	61	18 (58.1)	43 (63.2)	0.241	0.624
Female	38	13 (41.9)	25 (36.8)		
Tumor classification^**§**^					
T1-2	9	5 (16.1)	4 (5.9)	‒	0.134
T3-4	90	26 (83.9)	64 (94.1)		
Lymph node metastasis					
Absent	64	25 (80.6)	39 (57.4)	5.055	**0.025**
Present	35	6 (19.4)	29 (42.6)		
Distant metastasis^**§**^					
Absent	92	30 (96.8)	62 (91.2)	‒	0.429
Present	7	1 (3.2)	6 (8.8)		
TNM stage(AJCC)					
Stage I-II	61	25 (80.6)	36 (52.9)	6.910	**0.009**
Stage III-IV	38	6 (19.4)	32 (47.1)		

Note: Values in parentheses indicate percentage values. The bold number represents the *P* values with significant differences. ^**§**^ represents Fisher's exact probability test.

### PHLDA2 knockdown inhibits proliferation of CRC cells

**Figure 2:**
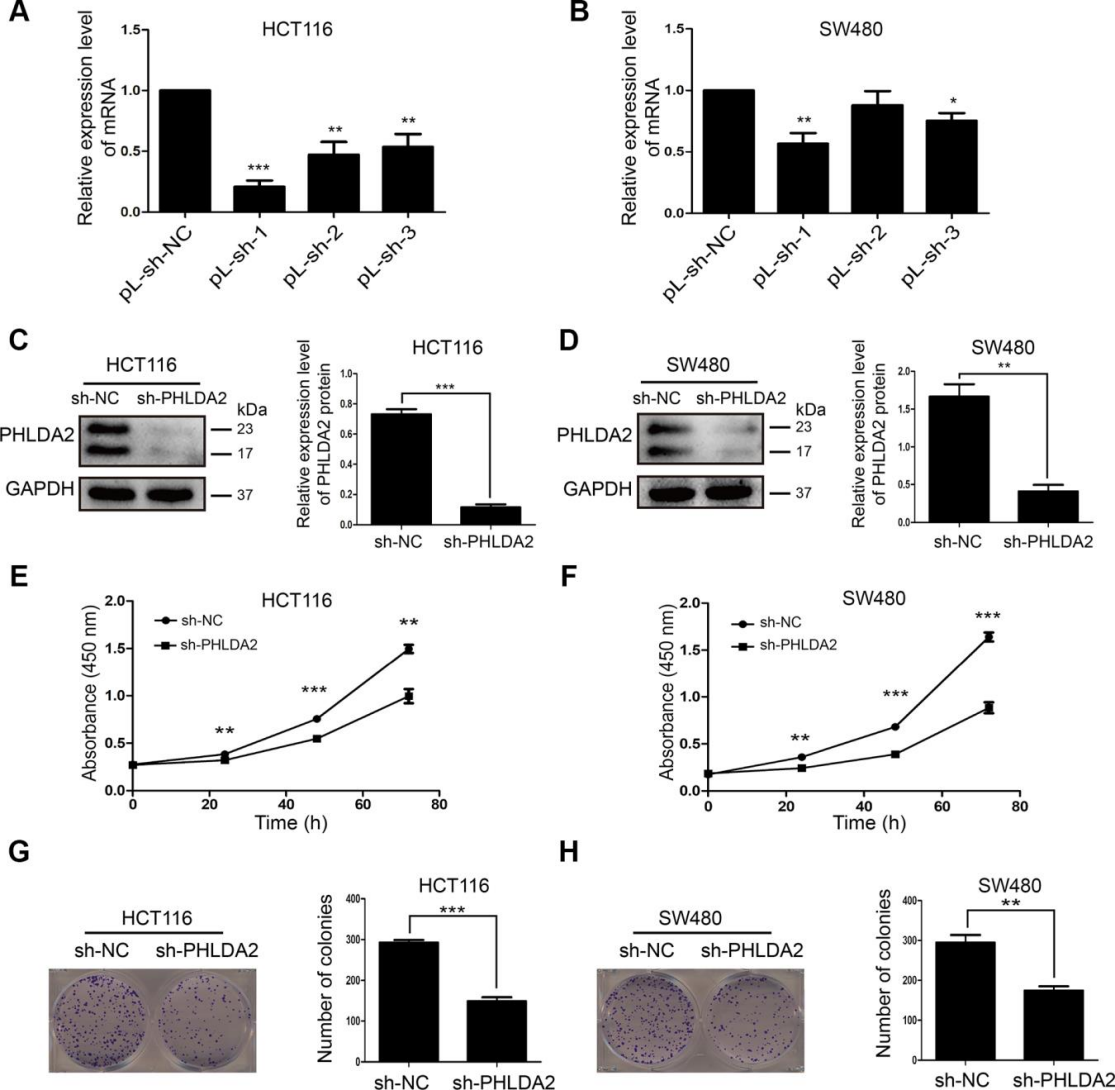
**Inhibition of PHLDA2 inhibits CRC cell proliferation.** (**A**, **B**) RT-qPCR was used to assess the knockout efficiency of three pLVX-sh-PHLDA2 knockdown fragments in HCT116 and SW480 cells. (**C**, **D**) Western blot was used to assess the knockout efficiency of the sh-PHLDA2 lentivirus vector in HCT116 and SW480 cells. (**E**–**H**) Cell Counting Kit-8 (CCK8) and colony formation assays were used to assess cellular proliferation. Data are shown as mean ± SD; *P < 0.05, **P < 0.01, and ***P < 0.001.

Since we selected HCT116 and SW480 for *in vitro* studies, we generated stably-transfected cells with low PHLDA2 expression. The highest knockout efficiency was exhibited by pL-sh-1 (Figure 2A, 2B). Lentivirus vector (sh-PHLDA2) strongly inhibited PHLDA2 protein levels in HCT116 (*P* < 0.001, Figure 2C) and SW480 (*P* < 0.01, Figure 2D) cells. To investigate the effect of PHLDA2 in CRC cells, we evaluated cell proliferation. The CCK8 assay demonstrated that low-expression of PHLDA2 inhibited HCT116 (*P* < 0.01, Figure 2E) and SW480 (*P* < 0.01, Figure 2F) cell growth. Colony formation assays revealed low-expression of that PHLDA2 suppresses the proliferation of HCT116 (*P* < 0.001, Figure 2G) and SW480 (*P* < 0.01, Figure 2H) cells. These results demonstrate that low-expression of PHLDA2 inhibits the proliferation of CRC cells.

### PHLDA2 knockdown in CRC cells inhibits migration and invasion by downregulation of EMT

**Figure 3:**
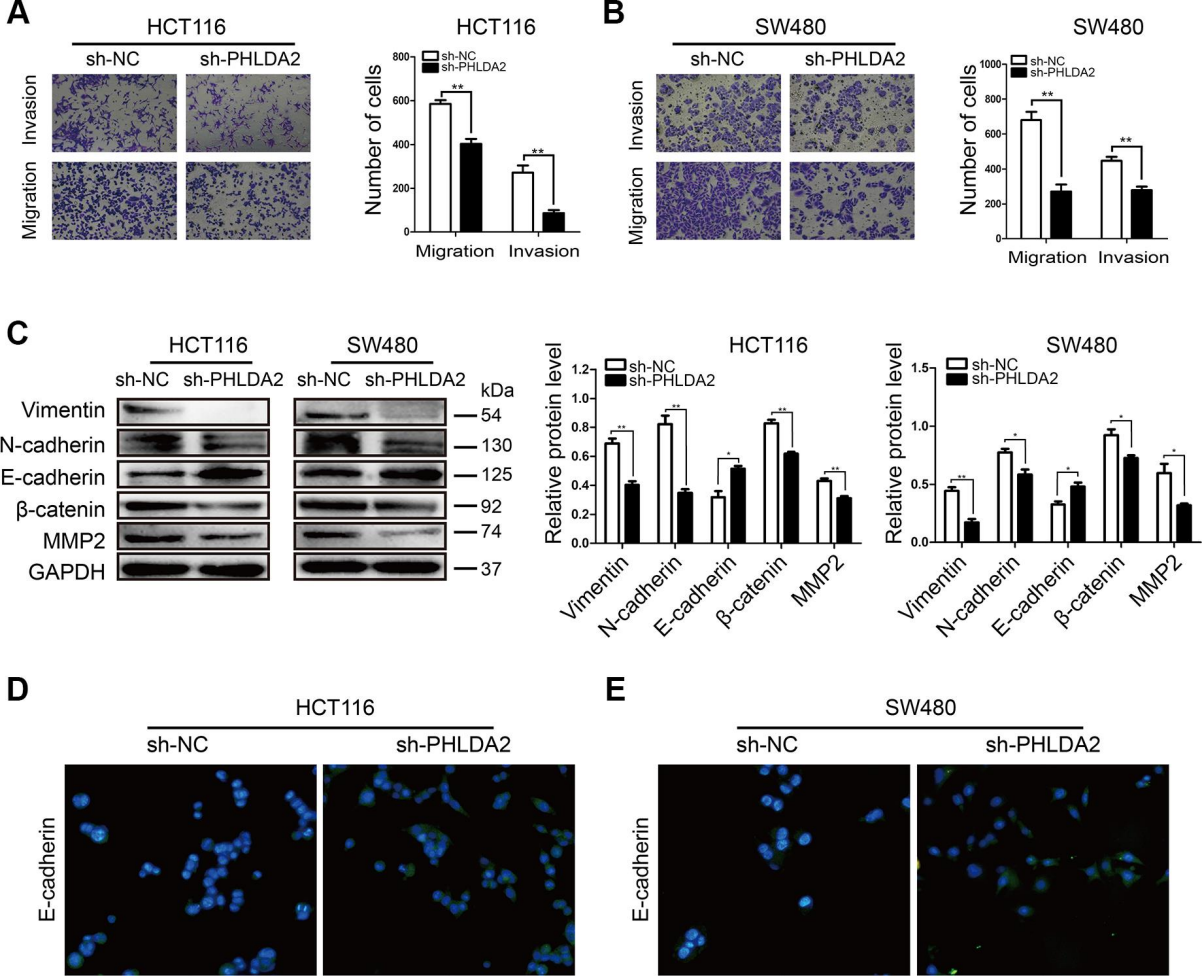
**Low PHLDA2 expression inhibits CRC cellular migration and invasion by down regulation of EMT.** (**A**, **B**) Invasion and migration of HCT116 and SW480 cells were measured by flow cytometry. (**C**) Western blots showing the levels of EMT-associated proteins; Vimentin, N-cadherin, E-cadherin, β-catenin, and MMP2. (**D**, **E**) Protein expression and sub-localization of E-cadherin as visualized by immunofluorescence. Data are shown as mean ± SD; *P < 0.05, ** *P*< 0.01, and ***P < 0.001.

To assess the effect of PHLDA2 on migration and invasion of CRC cells, we performed Transwell and Matrigel assays. Invasion and migration by HCT116 (*P* < 0.01, Figure 3A) and SW480 (*P* < 0.01, Figure 3B) cells were reduced by sh-PHLDA2. Sh-PHLDA2 also reduced the levels of EMT-related proteins including; N-cadherin, Vimentin, β-catenin, and MMP2. In contrast, sh-PHLDA2 increased E-cadherin levels in CRC cells (Figure 3C–3E). Therefore, a reduction in PHLDA2 levels inhibits invasion and migration by CRC cells through effects on EMT.

### PHLDA2 knockdown induces cell cycle arrest and increases apoptosis of CRC cells

**Figure 4:**
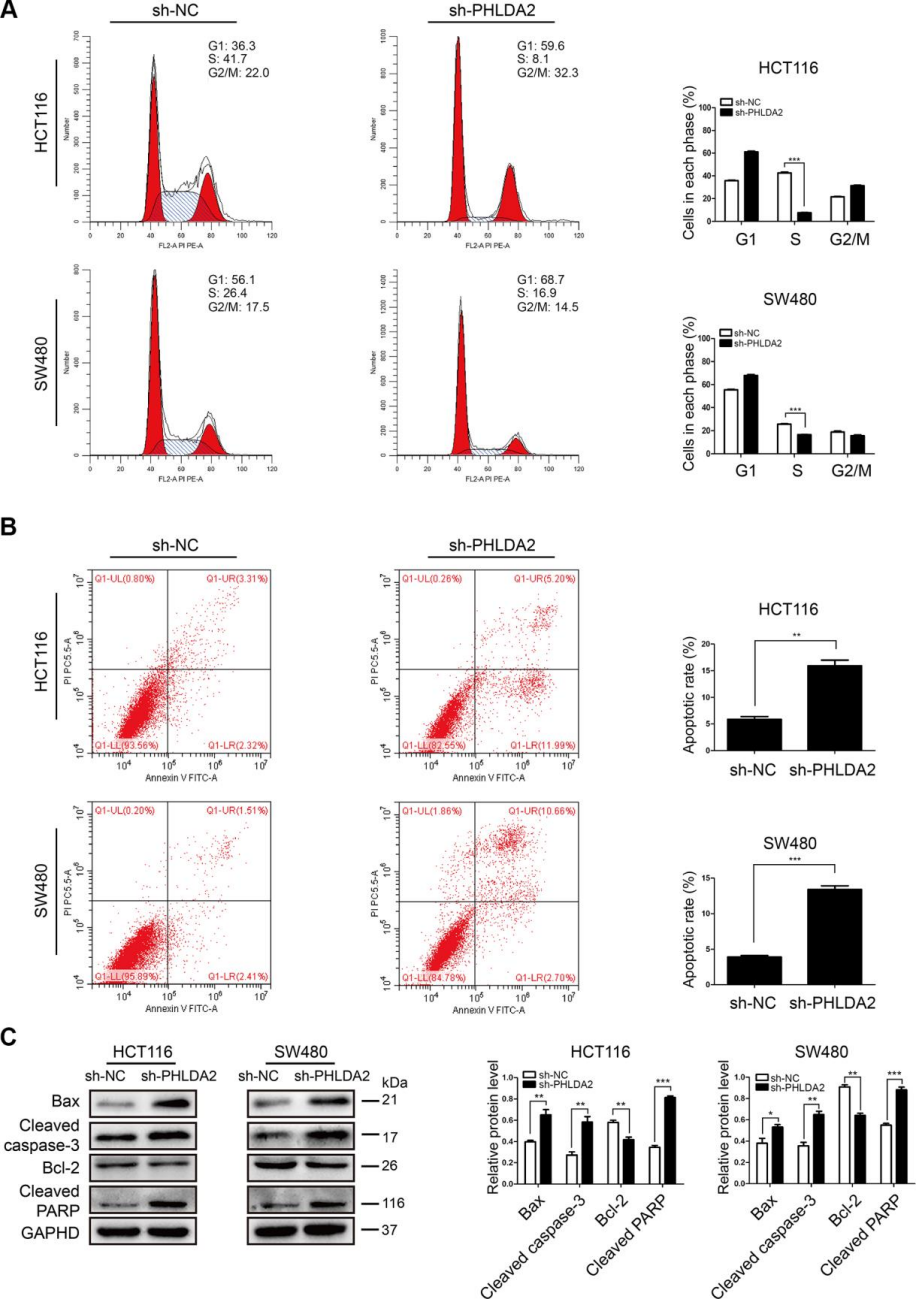
**Inhibition of PHLDA2 induces cell cycle arrest in G1/S phase and increases apoptosis in CRC cells.** (**A**, **B**) Apoptosis and cell cycle phase of transfected cells as measured by flow cytometry. (**C**) Western blots showing the levels of apoptosis-associated proteins (Cleaved caspase-3, Cleaved PARP, Bax, and Bcl-2) were assessed. Data are shown as mean ± SD; *P < 0.05, ** *P*< 0.01, and ***P < 0.001.

Analysis of cell cycle distribution and apoptosis of HCT116 and SW480 cells showed that knockdown of PHLDA2 inhibition stalled cells in G1/S phase (*P <* 0.001, Figure 4A) and increased apoptosis (Figure 4B). Western blot demonstrated an increase in the expression of apoptosis-related proteins, including Cleaved caspase-3, Cleaved PARP, and Bax. Furthermore, sh-PHLDA2 transfection decreased Bcl-2 levels in CRC cells (Figure 4C). These results suggest that inhibition of PHLDA2 induces cell cycle arrest and apoptosis of HCT116 and SW480 cells.

### Low expression of PHLDA2 enhances autophagic flux in CRC cells

**Figure 5:**
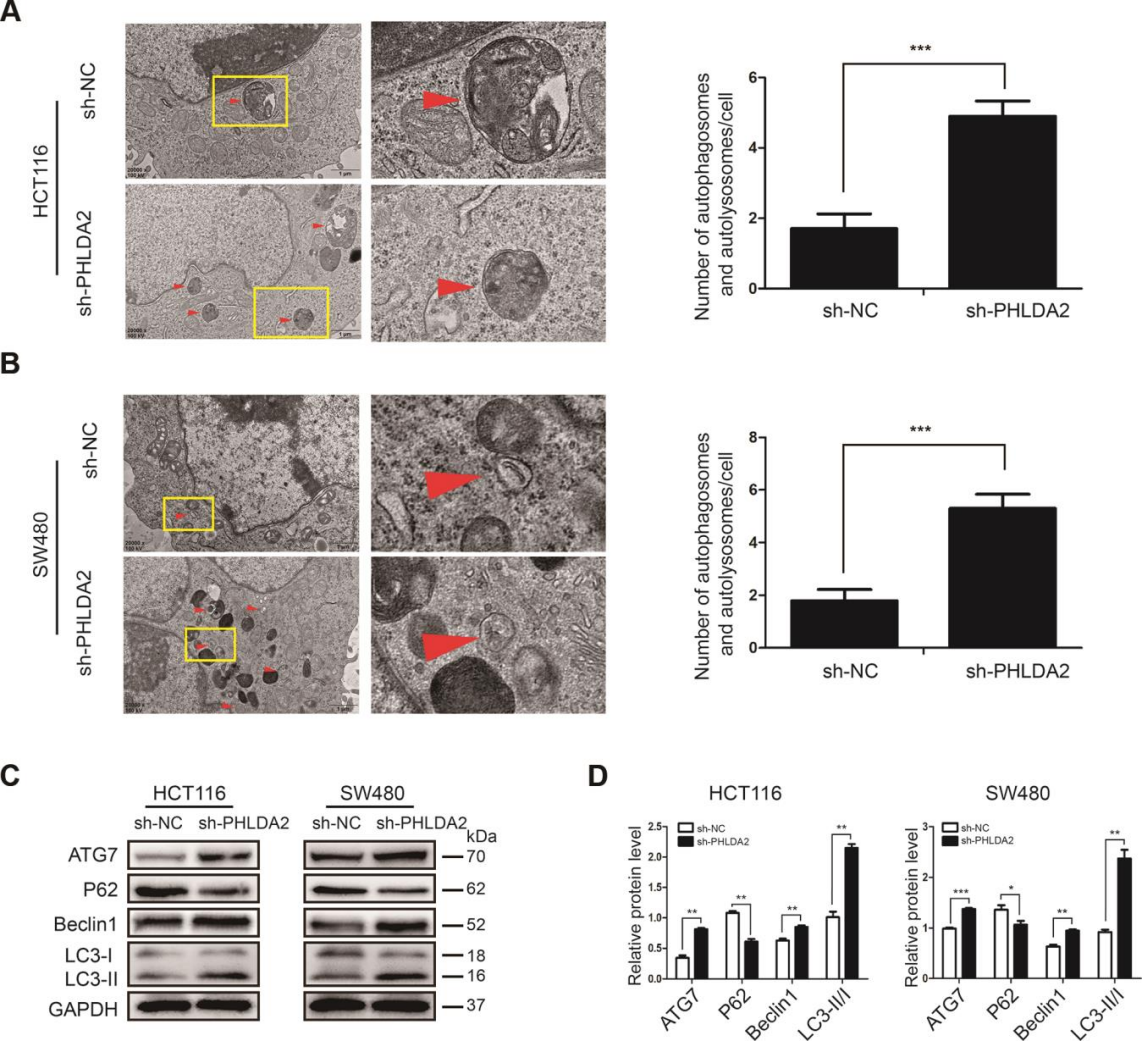
**Low PHLDA2 expression enhances autophagic flux in CRC cells.** (**A**, **B**) We used TEM (×20,000) to image HCT116 and SW480 cells and quantified the number of autophagic vacuoles (autophagosomes and autolysosomes) in subsets of 10 randomly-selected cells of each type. Red arrows, autophagic vacuoles; *P<0.05, ***P<0.001. (**C**, **D**) Expression of autophagy-associated proteins ATG7, Beclin1, LC3, and P62. Data are shown as mean ± SD; *P < 0.05, **P < 0.01, and ***P < 0.001.

We found that PHLDA2 expression correlates negatively with apoptosis of CRC cells. Autophagy is a hallmark of cellular apoptosis; therefore, we assessed the effect of PHLDA2 on CRC cellular apoptosis. We used Transmission electron microscopy (TEM) to examine CRC cells and found stably-transfected with sh-PHLDA2 increased numbers of autophagosomes and autolysosomes in HCT116 (Figure 5A**)** and SW480 (Figure 5B**)** cells. Western blot analyses demonstrated an accumulation of ATG7, Beclin1, and lipid-bound LC3-II in cells with low PHLDA2 levels (Figure 5C, 5D), indicating an increase in the synthesis of autophagosomes. We also measured the levels of P62, a substrate of autophagy. As expected, inhibition of PHLDA2 decreased the expression of P62 protein (Figure 5C, 5D). Taken together, these results suggest that low PHLDA2 levels induce autophagy in CRC cells.

### PHLDA2-mediated autophagy contributes to apoptosis of CRC cells induced by low PHLDA2 expression

**Figure 6:**
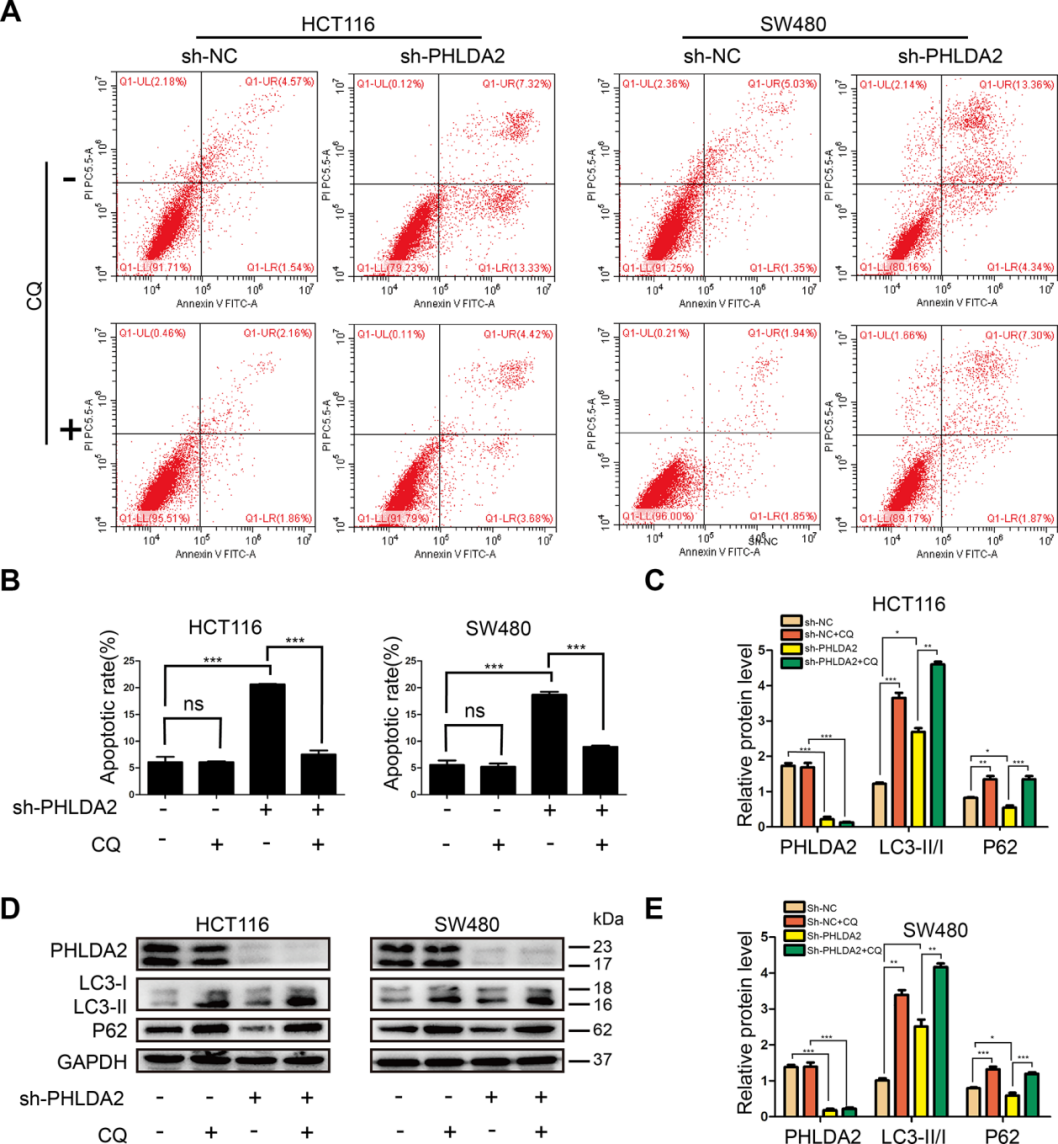
**PHLDA2-mediated autophagy contributes to apoptosis induced by PHLDA2 inhibition in of CRC cells.** (**A**, **B**) We used flow cytometry to detect cellular apoptosis with or without PHLDA2 inhibition, as well as with or without autophagy suppression by chloroquine (CQ). (**C**–**E**) Protein levels of LC3 and p62 were assessed by western blot with or without PHLDA2 inhibition, as well as with or without autophagy suppression by CQ. Data are shown as mean ± SD; ns: no significant difference; *P < 0.05, **P < 0.01, and ***P < 0.001.

Inhibition of PHLDA2 increased autophagy (Figure 5A, 5B) and induced apoptosis (Figure 4B, 4C) of CRC cells. To determine whether PHLDA2-induced inhibition of apoptosis was related to autophagy, we used flow cytometry to assess cellular apoptosis with or without PHLDA2 inhibition, and with or without autophagy suppression by chloroquine (CQ). Results showed that inhibition of PHLDA2 increased apoptosis of HCT116 and SW480 cells. No obvious cytotoxicity was found in control cells treated with CQ alone (Figure 6A, 6B). CQ blocked autophagic flux (Figure 6C–6E) and partially rescued apoptosis in cells with low PHLDA2 levels (Figure 6A, 6B). These data demonstrate that PHLDA2-induced apoptosis in CRC cell lines is associated with an increased autophagic flux.

### The effect of PHLDA2 on autophagy and EMT is partially dependent upon the PI3K/AKT signaling pathway

**Figure 7:**
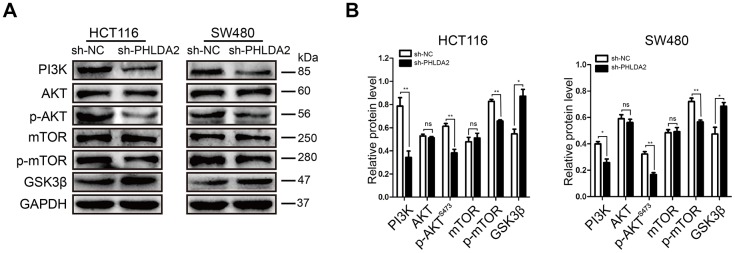
**The effects of PHLDA2 on autophagy and EMT are partially dependent upon the PI3K/AKT signaling pathway in CRC cells.** (**A**, **B**) Levels of PI3K, AKT, p-AKT^S473^, mTOR, p-mTOR, and GSK3β proteins were assessed in stably-transfected HCT116 and SW480 cells. Data are shown as mean ± SD; ns: no statistically significant difference; *P < 0.05, **P < 0.01.

A previous study reported that PHLDA2 inhibited the phosphorylation of AKT [6]. In view of the contributions of the PI3K/AKT pathway to autophagy and EMT, we measured PI3K, AKT and p-AKT^S473^ levels in HCT116 and SW480 cells stably-transfected with sh-PHLDA2 and sh-NC. Our results showed that PHLDA2 inhibition decreased the levels of PI3K and p-AKT^S473^, but had no effect on AKT levels (Figure 7A, 7B), suggesting that the PI3K/AKT pathway may be involved in PHLDA2-induced autophagy and EMT. To further investigate the underlying molecular basis for PHLDA2-mediated autophagy and EMT, we measured the levels of mTOR, p-mTOR, and GSK3β. We found that low levels of PHLDA2 promoted higher levels of GSK3β and lower levels of p-mTOR in HCT116 and SW480 cells, but did not affect mTOR levels (Figure 7A, 7B). These results indicate that PHLDA2-induced autophagy and EMT partially depend on the PI3K/AKT/mTOR and PI3K/AKT/GSK3β pathways, respectively.

### *In vivo* data

### PHLDA2 modulates tumorigenesis

**Figure 8:**
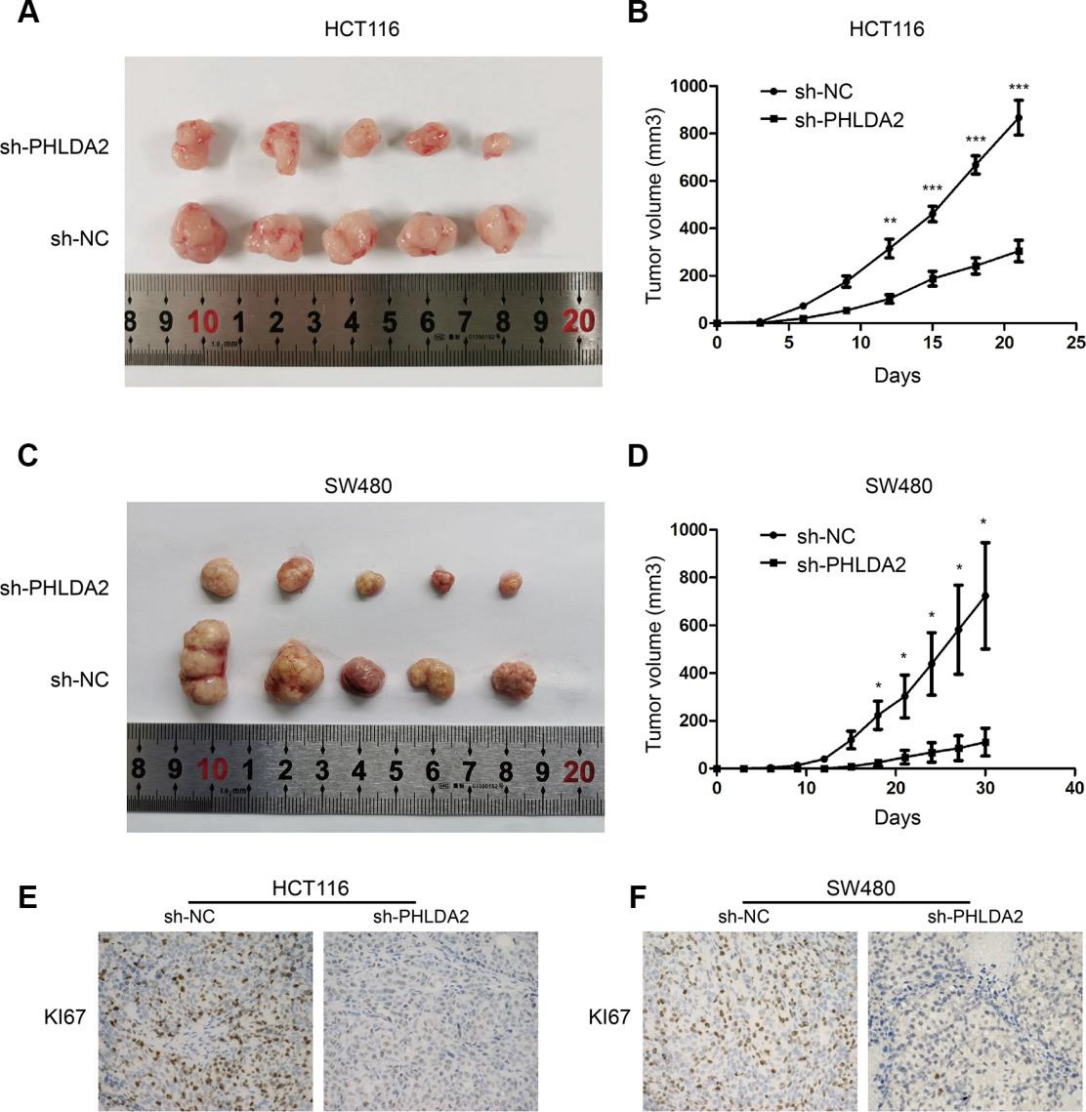
**PHLDA2 modulates tumorigenesis autophagy and apoptosis in vivo.** (**A**–**D**) The volume and growth curve of nude mouse tumors. (**E**, **F**) Levels of KI67 proteins in tumors from nude mice. *P < 0.05, ** *P*< 0.01, and ***P<0.001.

In the above *in vitro* experiments, we demonstrated that PHLDA2 promoted CRC cell growth, inhibited apoptosis, promoted EMT, and inhibited autophagy partly through the PI3K/AKT signaling pathway. We next tested the role of PHLDA2 *in vivo*. Stably-transfected HCT116 and SW480 cells were injected subcutaneously into the left flank of nude mice. We found that inhibition of PHLDA2 reduced the proliferation of HCT116 and SW480 cells in this mouse tumor model (Figure 8A–8D) and inhibited the expression of KI67 protein (Figure 8E, 8F). These results are consistent with the finding that inhibition of PHLDA2 inhibits cell proliferation *in vitro* (Figure 2E–2H), suggesting that PHLDA2 promotes CRC progression *in vivo* and *vitro*.

## DISCUSSION

Cancer development is multi-factorial in nature with multiple underlying contributing causes, such as heredity, diet, lifestyle, and gene modifications, among others [16–19]. Therefore, the identification of genes that promote the occurrence and development of CRC could benefit diagnosis and treatment. PHLDA2 is an imprinted gene associated with several cancers. Imprinted genes vary in their tissue-specificity and in mono-allelic expression with inhibition of tumorigenesis demonstrated in osteosarcoma [20]. Furthermore, PHLDA2 promotes tumor formation in triple-negative breast cancer [7] and in pancreatic ductal adenocarcinoma [8]. However, the effects of PHLDA2 on CRC is not known. In this study, we demonstrated that PHLDA2 is overexpressed in CRC tissue and that high PHLDA2 levels correlate with lymphatic metastasis and TNM stage. No relationship was found for distant metastasis in the limited number of samples we analyzed. On the other hand, low expression of PHLDA2 inhibited the proliferation, migration, and invasion of CRC cells while promoting apoptosis. Strong PHLDA2 inhibition induced autophagy and inhibited EMT, partly through the PI3K/AKT/mTOR and PI3K/AKT/GSK3β signaling pathways, respectively. EMT partially contributed to PHLDA2-induced cancer metastasis. in addition, autophagy, which likely contribute to the PHLDA2-induced apoptosis we observed for CRC cells.

EMT is a widely-known biological process that is essential to cancer progression since it transforms epithelial cells into mesenchymal cells and thereby promotes metastasis [21]. Here, we demonstrated that low levels of PHLDA2 inhibit the invasion and migration of CRC cells (Figure 3A, 3B**)**, which suggests that PHLDA2 may promote EMT. High levels of β-catenin, E-cadherin, N-cadherin, Vimentin, and MMP2 are markers for EMT [22]. By measuring their levels, we demonstrated that low expression of PHLDA2 partly restrained EMT (Figure 3C–3E**)**. These results suggest that EMT contributes to PHLDA2-mediated invasion and migration of CRC cells.

Autophagy is a lysosomal degradation pathway involved in a number of harmful conditions [12]. Cancer cells face more environmental and intrinsic metabolic stressors than normal cells, and are therefore, more reliant on autophagy [23]. Many reports have shown a close relationship between autophagy and cancer cell apoptosis [24, 25]. PHLDA1, a gene homologous to PHLDA2, inhibits autophagy in neuroblastoma [26] and promotes autophagy in breast cancer cells [27]. In addition, PHLDA2 has been reported to activate autophagy in osteosarcoma cells [6], which suggests that PHLDA2 may regulate autophagy in CRC cells. Here, we observed that low-expression of PHLDA2 induced autophagy in CRC cells (Figure 5A–5D). As mentioned above, autophagy has dynamic and complicated functions in cancer [12, 23, 28, 29], regulating cellular apoptosis in stressful environments. Therefore, we investigated the relationship between autophagy and apoptosis in CRC cells. This study showed that CQ, as an autophagy inhibitor, partially reduced the apoptosis induced by low levels of PHLDA2 in CRC cells (Figure 6A, 6B). Thus, PHLDA2 inhibition increased apoptosis in CRC cells partly through the activation of autophagy. However, the mechanistic basis for PHLDA2 low-expression activation of autophagy and inhibition of EMT in CRC cells is unclear and requires further investigation.

The PI3K/AKT signaling pathway is an important cancer cell pathway. Akt inactivates the proapoptotic factors Bad and procaspase-9 by phosphorylation to regulate apoptosis, and inhibits GSK3 to induce cell cycle progression via regulation of RB hyperphosphorylation and inactivation [30]. Akt also phosphorylates p21 and inhibits its antiproliferative effects by retaining it within the cytoplasm [31]. PI3K is a lipid kinase and converts PIP2 into PIP3. PIP3 is a second messenger essential for the translocation of AKT to the plasma membrane where it is phosphorylated and activated by PDK1 and PDK2 [32]. mTOR is one of the key proteins in the PI3K/AKT/mTOR pathway [33]. Activation of AKT promotes mTOR activity by suppressing the repressive action of tuberous sclerosis complex (TSC) through phosphorylation of TSC2 at serine residue 939 and binding of 14-3-3 protein [34]. In addition, autophagy-related proteins, such as ULK1, ATG13, AMBRA1, and ATG14L promote autophagy initiation and autophagosome nucleation. mTOR inhibits autophagy by phosphorylation of these autophagy-related proteins [35]. The results of our study indicate that knockdown of PHLDA2 partly decreases the expression of PI3K, p-AKT^S473^ and p-mTOR, promoting autophagy. The PI3K/AKT pathway plays a crucial role in EMT with p-AKT phosphorylating GSK3β at Ser9. This phosphorylation of GSK3β inhibits kinase activity, which down regulates both T-β-catenin and N-β-catenin [36]. β-catenin is an important regulator of EMT [37]. Therefore, inhibition of PHLDA2 decreases the expression of PI3K, p-AKT^S473^, and p-mTOR, and increases the expression of GSK3β, which activates autophagy and inhibits EMT. Importantly, low levels of PHLDA2 inhibited tumor growth *in vivo*.

In conclusion, we demonstrated that PHLDA2 is a CRC tumor promoter. Low level of PHLDA2 inhibited the proliferation, invasion, and migration, as well as enhanced apoptosis of CRC cells *in vitro*. Similarly, silencing PHLDA2 reduced tumor growth *in vivo*. In addition, inhibition of PHLDA2 inhibited EMT and induced autophagy partly via the PI3K/AKT/mTOR and PI3K/AKT/GSK3β signaling pathways, respectively. Furthermore, low levels of PHLDA2 induced autophagy, which contributed to apoptosis in CRC cells. Low expression of PHLDA2 also inhibited EMT, contributing to suppression of invasion and migration in CRC cells. We anticipate that this study will provide a basis for diagnosis and treatment of CRC. However, this study has limitations, including a lack of data for PHLDA2 expression in normal colorectal cells, other protein inhibitors of the PI3K/AKT pathway were not assessed, and the limited number of CRC samples that were evaluated. These limitations will be addressed in the future.

## MATERIALS AND METHODS

### Patient specimens

In this study, 29 fresh CRC samples and paired adjacent benign tissue samples were randomly collected from the First Affiliated Hospital of Chongqing Medical University (Chongqing, China from June to December, 2017). RNA was extracted and detected by reverse transcription-quantitative polymerase chain reaction (RT-qPCR). Ninety-nine paraffin sections of CRC and 27 normal tissues were obtained from the Department of Pathology of the First Affiliated Hospital of Chongqing Medical University (June, 2012 to September, 2013) and were used to measure protein levels. Informed consent was signed by all patients, and the study was supported by the Ethics Committee of the First Affiliated Hospital of Chongqing Medical University.

### Cell culture and transfection

Human CRC cell lines (Caco2, HT29, SW480, LOVO, HCT116, and SW620) were purchased from the American Type Culture Collection (Manassas, VA, USA). All cells were cultured in RPMI-1640 (Gibco, CA) supplemented with 10% fetal bovine serum (FBS) (PAN, Germany) with standard conditions at 37 °C, 5% CO2, and a humidified atmosphere. To inhibit autophagy, cells were treated with 10 nm chloroquine (CQ, Sigma-Aldrich, USA) for 12 h. Short hairpin RNA (shRNA) in the pLVX-shRNA1 plasmid vector targeting PHLDA2; (pLVX-sh-PHLDA2; sh1, 5‘-GCAAGTACGTGTACTTCACCA-3’; sh2, 5‘-GACCACAAGGAG ATCGACTTC-3’; sh3, 5‘-GCGCTCATCGATTTCCAGAAC-3’) and its negative control pLVX-shRNA1 (sh-NC) were purchased from GeneCopeia, Inc. (Rockville, MD, USA). The HCT116 cells were transfected using Lipofectamine 2000 (Invitrogen Preservation, Carlsbad, CA, USA) according to the manufacturer's protocol. The plasmid with the highest knockdown efficiency (pL-sh-1) and the negative control plasmid (pL-sh-NC) were selected and packaged into lentivirus (sh-PHLDA2 and sh-NC) according to the manufacturer's protocol, and then transfected into HCT116 and SW480 cells. Knockdown efficiency was assessed by western blot (WB).

### RNA extraction and reverse transcription-quantitative polymerase chain reaction (RT-qPCR)

RT-qPCR experiments were performed as previously described [38]. The primers used in this study were: PHLDA2, forward, 5'-GCGAGGGCGAGTTGGAGAA-3′, and reverse, 5'-CCACCTTGAGGATGGAGTGGAA-3′, GAPDH, forward, 5'-TGACTTCAACAGCGACA CCCA-3 ' and reverse, 5'-CACCCTGTTG CTGTAGCCAAA-3'. The qPCR assays were performed with TB Green™ Premix Ex Taq (Takara Biotechnology Co., Ltd.). The qPCR conditions were as follows: Pre-denaturation at 95 ^o^C for 30 s, followed by 40 cycles at 95 ^o^C for 5 s, with annealing and extension at 60 ^o^C for 30 s. GAPDH was uses as the internal reference. The relative mRNA expression of target genes was measured by the 2-ΔΔCq method [39].

### Immunohistochemistry (IHC) and immunofluorescence (IF)

IHC staining of CRC tissue samples and xenografted tumors was performed as described previously [40]. Sections were incubated with primary antibody PHLDA2 (Bs-6884R; 1:100), KI67 (ab15580; 1:200). The tissue sections were imaged with a Leica microscope imaging system (Leica Microsystems GmbH, Wetzlar, Germany). Results were analyzed as previously described [41].

For IF staining, cells were cultured on glass coverslips in 6-well plates, fixed with 4% paraformaldehyde, treated with 0.5% Triton X-100 (Beyotime, China), blocked with 1% goat serum albumin, and incubated with primary antibody E-cadherin (20874-1-AP, 1:50) overnight at 4 °C. After treatment with secondary antibody (SA00009-2 Cy3-conjugated goat anti-rabbit), cell nuclei were stained with DAPI (Sigma-Aldrich; Merck KGaA). Images were captured with a fluorescence microscope (× 400, Leica Microsystems GmbH).

### Cell proliferation assay

Cell proliferation was measured with a Cell Counting Kit-8 (CCK8) [42]. Briefly, stably-transfected HCT116 and SW480 cells were inoculated into 96-well plates at a density of 2,000 cells per well and cultured at 37 °C in 5% CO_2_. The CCK-8 reagent was added at 0, 24, 48, and 72 h. Absorbance at 450 nm was detected with a microplate reader (Bio-Rad Laboratories, Inc.).

The colony formation assay was performed as previously described [43]. Briefly, HCT116 and SW480 cells were inoculated into 6-well plates at a density of 500 per well and cultured for two weeks. The cells were fixed with 4% paraformaldehyde for 30 min, stained with 1% crystal purple for 20 min, and washed three times with PBS. The numbers of clones (> 50 cells/colony) were counted with an inverted phase contrast microscope (Leica, Wetzlar, Germany).

### Cell migration and invasion assays

Cell migration and invasion assays were performed with Transwell chambers (Corning Inc., Corning, NY, USA). For the migration assay, the stably-transfected cell lines (HCT116 and SW480) were digested with trypsin and suspended (2.5 × 10^5^ cells/ml) in RPMI-1640 medium. Cell suspensions (200 μl) were added to the upper chamber, and 700 μl of RPMI-1640 containing 10% FBS added to the lower chamber. After 48 h, the migrated cells were fixed with 4% paraformaldehyde and stained with 0.1% crystal violet for 20 min at room temperature. Subsequently, the cells from five random fields were counted with a microscope (× 200 magnification; Leica Microsystems GmbH, Wetzlar, Germany). For the invasion assay, Matrigel (BD Biosciences) was diluted with RPMI-1640 at a 1:8 ratio. A 100 μl aliquot of the mixture was added to the upper chambers and incubated at 37 ^o^C for 5 h to solidify. A 100 μl RPMI-1640 aliquot containing 1 × 10^5^ cells was added to the upper chambers with the subsequent steps identical to the migration assay.

### Cell cycle and apoptosis assays

For the cell cycle assay, cell culture supernatants were discarded, stably-transfected cells (HCT-116 and SW480) were digested with trypsin and washed with PBS. Then, the cells were fixed in 70% iced ethanol, prepared in advance for 24 h at 4 ^o^C. After centrifugation, RNase A (10 mg/ml; Sigma-Aldrich; Merck KGaA) was added to the cells and incubated for 30 min at 37 ^o^C in 5% CO_2_. The cells were then stained with propidium iodide (PI; Beyotime Institute of Biotechnology, Shanghai, China). Cell-cycle was detected by flow cytometry (BD Biosciences, San Jose, CA, USA) and analyzed by FlowJo version 7.6 software (FlowJo LLC, Ashland, OR, USA).

For cellular apoptosis, cell culture supernatants were collected and stably transfected cells (HCT-116 and SW480) were washed, digested, centrifuged, and diluted in PBS. Cells were stained with PI (BD Pharmingen; BD Biosciences) and annexin V-fluorescein isothiocyanate for 15 min in the dark. Apoptosis was examined by flow cytometry (BD Biosciences, San Jose, CA, USA). The apoptotic rate was assessed with BD FACS software.

### Transmission electron microscopy (TEM)

For TEM, cells were washed, digested, centrifuged, collected, and fixed with 2.5% glutaraldehyde. Stably-transfected cells (HCT-116 and SW480) were embedded in Epon 812 resin and dehydrated in various concentrations of acetone. The specimens were cut into 1 μm slices and stained with sodium acetate and lead citrate. The cells were observed with a transmission electron microscope (JEM-1400Plus; JEOL, Tokyo, Japan). The number of autophagic vacuoles, including autophagosomes and autolysosomes, in each cell was quantified in 10 randomly-selected cells from each group [44].

### Western blot (WB) analysis

Proteins were extracted from stably-transfected HCT-116 and SW480 cells, as well as from nude-mice tumors, and were separated by sodium dodecyl sulphate-polyacrylamide gel electrophoresis (SDS-PAGE). The proteins were transferred to polyvinylidene difluoride (PVDF) membranes. The membranes were blocked with 5% skim milk for 1-2 h and incubated at 4 °C overnight with primary antibodies, and then with goat anti-rabbit or anti-mouse antibodies (1:5000; Abbkine, Redlands, CA, USA) at 37 °C for 1 h. The membranes were examined with a Bio-Rad gel imaging system (Bio-Rad, Hercules, CA, USA) with a WB kit (Advansta, Menlo Park, USA). Results were analyzed with Image J software (version 6.0; National Institutes of Health, Bethesda, MD, USA), normalized to GAPDH. The primary antibodies used for WB analysis included: PHLDA2 (14661-1-AP, 1:800), GAPDH (60004-1-Ig, 1:5000), E-cadherin (20874-1-AP, 1:5000), N-cadherin (22018-1-AP, 1:3000), and Vimentin (10366-1-AP, 1:2000) from Proteintech Group Inc. (Chicago, IL, USA); PI3K (YM3800, 1:1000), MMP2 (YT2798, 1:1,000), GSK3β (YT2082, 1:1,000), and β-catenin (YM6676, 1:1,000) purchased from Immunoway Biotechnology Company (Plano, TX, USA); P62 (ab109012, 1:5000), Bax (ab32503, 1:5000), Bcl-2 (ab32124, 1:2000), ATG7 (ab52472, 1:5000), Beclin-1 (ab207612, 1:2000), p-AKT^Ser473^ (ab81283, 1:3000), AKT (ab8805, 1:1000), p-mTOR (ab109268, 1:1000), Cleaved caspase-3 (ab2302, 1:500), LC3 (ab51520, 1:3000), and mTOR (ab2732, 1:2000) from Abcam, Inc. (Cambridge, UK); cleaved PARP (#5625, 1:1000) from Cell Signaling Technology Inc. (Danvers, MA, USA).

### *In vivo* tumorigenesis

Four week-old male BALB/c nude mice were purchased from Huafukang Biotechnology (Beijing, China) and housed in the Animal Experimental Center of Chongqing Medical University (Chongqing, China). All animal experiments were performed with the approval of the Ethical Committee of Chongqing Medical University with the use of experiment animals in accordance with the Guide for Care and Use of Laboratory Animals (US National Institutes of Health). The mice were randomized into four groups with five mice per group (HCT116-sh-NC, HCT116-sh-PHLDA2, SW480-sh-NC and SW480-sh-PHLDA2). Stably-transfected HCT116 and SW480 cells were digested, centrifuged, washed twice with PBS, and diluted in a 100 μl suspension with PBS containing 1×10^6^ cells. The suspensions were injected subcutaneously into the left flank of the nude mice to induce formation of xenografted tumors. The nude mice were maintained with normal feed and water for 21-30 days and then tumors were resected for analysis. Tumor volume was measured every three days using a Vernier caliper. Tumor volume was calculated using the formula: V = (length × width^2^)/2. Finally, the mice were anesthetized, euthanized, and humanely disposed of.

### Statistical analysis

Except where otherwise noted, experiments were repeated at least three times. Statistical analyses were performed using SPSS 22.0 software (Chicago, IL, USA). The data are presented as mean ± SD. Briefly, the Student’s *t*-test or analysis of variance (ANOVA) were used to analyze the data. The Chi-square test was used to analyze the relationships among PHLDA2 expression and clinical characteristics of CRC patients. *P*-values < 0.05 were considered statistically significant.
